# Jointly Feature Learning and Selection for Robust Tracking via a Gating Mechanism

**DOI:** 10.1371/journal.pone.0161808

**Published:** 2016-08-30

**Authors:** Bineng Zhong, Jun Zhang, Pengfei Wang, Jixiang Du, Duansheng Chen

**Affiliations:** Department of Computer Science and Technology, Huaqiao University, Xiamen, Fujian, 361021, China; Tianjin University, CHINA

## Abstract

To achieve effective visual tracking, a robust feature representation composed of two separate components (i.e., feature learning and selection) for an object is one of the key issues. Typically, a common assumption used in visual tracking is that the raw video sequences are clear, while real-world data is with significant noise and irrelevant patterns. Consequently, the learned features may be not all relevant and noisy. To address this problem, we propose a novel visual tracking method via a point-wise gated convolutional deep network (CPGDN) that jointly performs the feature learning and feature selection in a unified framework. The proposed method performs dynamic feature selection on raw features through a gating mechanism. Therefore, the proposed method can adaptively focus on the task-relevant patterns (i.e., a target object), while ignoring the task-irrelevant patterns (i.e., the surrounding background of a target object). Specifically, inspired by transfer learning, we firstly pre-train an object appearance model offline to learn generic image features and then transfer rich feature hierarchies from an offline pre-trained CPGDN into online tracking. In online tracking, the pre-trained CPGDN model is fine-tuned to adapt to the tracking specific objects. Finally, to alleviate the tracker drifting problem, inspired by an observation that a visual target should be an object rather than not, we combine an edge box-based object proposal method to further improve the tracking accuracy. Extensive evaluation on the widely used CVPR2013 tracking benchmark validates the robustness and effectiveness of the proposed method.

## 1. Introduction

Visual tracking is a fundamental task in computer vision applications, making it a key component of a real system. Consequently, it has been receiving a huge amount of attention and tremendous progress has been made in visual tracking over the past decades. However, designing robust tracking methods is still an open issue, especially considering various complicated variations that may occur in real-world scenes, e.g., partial occlusion, cluttered backgrounds, illumination changes, motion blur, scale variations, etc.

The performance of a tracking system mainly relies on the used feature representation technique. Typically, the feature representation is composed of two separate components, i.e., feature learning and selection. Towards these two components, a huge number of different methods for visual tracking have been proposed and a variety of features are utilized for modeling an object appearance model. Color or gray feature is widely used in the visual tracking literature to differ a target object from its surrounding backgrounds. In the famous mean shift-based tracking method [1], Comaniciu et al. employ a spatial-weighting color histogram for construing an object appearance model. Instead of using a fixed set of features, Collins et al. [2] propose an online feature ranking-based tracking method for continuously choosing the best set of features used to improve tracking performance. In [3], Possegger et al. propose a discriminative color model-based tracking method via mining distracting regions and adapting an object representation to suppress these regions. Zhang et al. [4] use a LAB color model to extract the features for visual tracking. Recently, Liang et al. [5] present a comprehensive survey on using color information for visual tracking from both the algorithm and benchmark perspectives.

Although color-based tracking methods can provide rich cues to effectively handle partial occlusion and pose variations in visual tracking, they may be sensitive to illumination variations and noises. Therefore, most modern visual tracking methods limit themselves to the more complicated features, e.g., Haar features, histogram of gradients (HoG), local binary pattern (LBP), etc. In addition to using raw pixel values, Henriques et al. [6] use HoG features to further improve tracking performance under a correlation filtering framework using the circulant matrices. Bertinetto et al. [7] propose a correlation filter-based tracking method via combining HoG features and a global color histogram. In [8], Zhang et al. propose a circulant sparse tracker which enables HoG features feasible for sparse representation-based trackers. Grabner and Bischof [9] propose an online adaboost-based tracking method using Haar features. In [10], Avidan propose an ensemble tracking method which uses Haar feature-based weak classifiers to adaptively construct a strong classifier. Takala et al. [11] combine color, LBP and motion features for multi-object tracking. Tong et al. [12] apply LBP features into visual tracking under the tracking-by-detection framework. Some key point-based descriptors are also used for visual tracking, e.g., SFIT and SURF etc. To obtain accurate boundaries of a target object, Fan et al. [13] use SIFT features as a short-term salient points to generate scribbles for robust matting. In [14], a lie algebra-based covariance matrix is utilized for visual tracking. In [15], Wang et al. propose an optimal appearance model-based tracking method, in which multiple cues are effectively integrated in the model. In [16], to effectively deal with multi-modal datasets, an online multi-modal non-negative dictionary learning method is used for visual tracking. However, one major drawback of the above handcrafted feature-based tracking method is that they are incapable to capture semantic information of targets, and not robust to significant appearance changes. On the other hand, the separated feature learning and selection component easily lead to the learned features not all relevant and noisy.

Recently, inspired by the success of deep learning in a variety of computer vision tasks [17–19], a large amount of deep leaning-based tracking methods have been proposed [20–28] for improve tracking performance. In [20], Fan et al. propose a convolutional neural network-based human tracking method which pre-learns the human-specific features during offline training. Wang and Yeung [21] propose a two-layer auto-encoder based tracker which is firstly pre-trained offline and then fine-tuned for an online tracking task. However, the discriminative power of the learned deep features may be limited due to the pre-training is performed in an unsupervised way. In [22], multiple convolutional neural networks are used for visual tracking. To further improve the discriminative power, some authors pre-train deep convolution networks on a large-scale image classification task (i.e., Imagenet) and then fine-tuned for a specific tracking task. By simultaneously using feature maps of multiple convolution layers from the VGG, Wang et al. [23] propose a fully convolutional neural network-based tracking method. In [24], Hong et al. employ a convolutional neural network which is pre-trained on Imagenet to predict saliency maps for online tracking. Ma et al. [25] firstly exploit feature maps from multiple convolution layers of a deep VGG to train multiple correlation filters. Then, the foreground heat maps estimated by the correlation filters are combined to provide robust tracking results. In [26], a multi-domain CNNs, composed of shared layers and multiple branches of domain-specific layers, is trained using a large set of videos with tracking ground truths for visual tracking. Each domain is trained for individual videos and each branch is used to classify a target object in each domain. In [27], CNN-based tracking method is proposed, in which a Hedge method is used to combine several CNN trackers from different CNN layers into a stronger one. To effectively transfer pre-trained deep features for online tracking, Wang et al. [28] present a sequential training method for convolutional neural networks. In [29], Tao et al. use a Siamese network for visual tracking. The Siamese network is pre-trained in a large and external videos to learn a matching mechanism. Despite achieving state-of-the-art tracking performance in recent benchmark evaluations [30, 31], most existing deep learning-based tracking methods still have some limitations due to blindly learn a representation using the majority of the learned high-level features.

In addition to focus on the feature representation, some authors have modeled an object appearance model using numerous advanced classifiers. The typical classifiers include correlation filters [6, 32, 33], ensemble learning [9, 34, 35], support vector machine [36–38], P-N learning [39], random forests [40], multiple instance learning [41], metric learning [42, 43], and sparse coding and low-rank matrices [44, 45] etc.

Recently, object proposal has made much progress for object detection [46–49] and segmentation. Inspired by this, several object proposal-based approaches [50–52] have also been proposed for robust visual tracking. In [50], visual tracking is viewed as an object proposal selection task. A fusion of detection confidence score, edges and motion boundaries is used to locate a target object. In [51], BING-based object proposal algorithm is adopted for visual tracking. To reduce a large amount of test space and provide a better training set for a tracker, Zhu et al. [52] employ an edge-ness based object proposal method for visual tracking. For a more comprehensive reviews on visual tracking methods, please refer to ([30, 31, 53] and [54]).

Despite achieving state-of-the-art tracking performance, most of the above visual tracking methods share a same basic assumption that the raw video sequences are clear. This assumption, however, may be too restrictive, especially under difficult conditions such as a complex real-world scene with significant noise and irrelevant patterns. In other words, most of the above tracking methods may fail if there is no good raw features to start with.

In this paper, to address the above-mentioned issues, we propose a novel unsupervised tracking algorithm via a point-wise gated convolution deep network (CPGDN) [55] that combines feature learning and feature selection coherently in a unified framework. Specially, the CPGDN is firstly pre-trained to automatically learn and select partially useful high-level abstractions from extracted image features on a Tiny image dataset [56]. Secondly, the CPGDN is further fine-tuned to adapt to a specific target object during online tracking. The proposed CPGDB-based tracker performs dynamic feature selection from the raw videos when the task-relevant patterns occur through a gating mechanism. Intuitively speaking, the model can adaptively focus on a variable subset of visible nodes corresponding to a specific target object instead of its surrounding backgrounds. Finally, to further improve tracking performance, we effectively incorporate an object proposal-based method (i.e., edge box-based proposals [46]) into the CPGDN-based tracker. This is inspired by an observation that most trackers are easily prone to locate on a non-object target (i.e., a background object or texture-less object) when the trackers have failed. Obviously, if a target object is non-object, the edge response is weak and the edge score is near zero. Therefore, we use an edge box-based proposal scoring function as a complementary cue to adjust the tracking results. We make an edge box based proposal score be negative if the edge box-based proposal method detects the non-object. A simple yet effective fusion schema is designed to combine the CPGDN model based score and the edge box-based proposal score. Extensive experiments on the CVPR2013 tracking benchmark [30], containing 50 sequences and 29 publicly available trackers, validate the robustness and effectiveness of the proposed tracking method. The main contributions of this work are three folds.

1. First, we design a unified feature learning and selection framework for visual tracking, in which the proposed tracking method is equipped with a CPGDN model trained end-to-end on the Tiny image dataset [56]. Consequently, the proposed tracking method is robust to the object appearance variations in video sequences.
2. Second, on the basis of the learnt object appearance model using the CPGDN model, we incorporate an edge box-based proposal scoring function into the object appearance model to further improve tracking performance.
3. Third, extensive experiments in the CVPR 2013 tracking benchmark [30] show that the proposed CPGDN-based tracker can achieve promising performance compared to the state-of-the-art trackers.

The rest of the paper is organized as follows. In Section 2, the proposed CPGDN-based tracking method is described in details. Then, we present an extensive evaluation of the proposed CPGDN-based tracker and demonstrate the experimental results in Section 3. Finally, we conclude remarks in Section 4.

## 2. The Proposed CPGDN-Based Tracking Method

In this section, we present our tracking method via a point-wise gated convolutional deep network (CPGDN), which can jointly performs feature learning and selection in a unified framework. Table 1 schematically show the proposed CPGDN-based tracking method under a particle filtering framework.

**Table 1:** Overview of the proposed CPGDN-based tracking method.

**Algorithm 1** Jointly Feature Learning and selection for Robust Tracking via a Gating Mechanism		
**Input:**		
	1. Pre-trained CPGDN filters {*w*^1^,*w*^2^,*w*^3^}	
	2. Initial target state *x*_1_.	
**Output:**		
	1. Estimated target states $x_{t}^{*}$.	
**Initialization:**		
	1. Initialize particles.	
	2. Randomly initialize the last full connect layer *w*^4^.	
	3. Collect positive samples $s_{1}^{+}$ and negative samples $s_{1}^{-}$.	
	4. Construct the CPGDN-based appearance model via fine-tuning using $s_{1}^{+}$ and $s_{1}^{-}$.	
**for t = 2 to the end of the video**		
**1. Prediction:** apply a prediction function in a particle filtering framework to obtain a set of candidate samples/particles ${\left\{c_{i}\right\}}_{i=1}^{N}$		
**2. Likelihood evaluation:**		
		(1) Calculate a detection score *f*_*T*_(*c*_*i*_) based on the CPGDN model for each particle ${\left\{c_{i}\right\}}_{i=1}^{N}$.
		(2) Calculate an edge box-based score *f*_*E*_(*c*_*i*_) using an edge box-based object proposal method for each particle ${\left\{c_{i}\right\}}_{i=1}^{N}$.
		(3) Find the optimal target state $x_{t}^{*}$ by Eq (6).
**3. Model updating:**		
		(1) Generate new positive and negative samples $s_{t}^{+}$ and $s_{t}^{-}$ according to the optimal target state.
		(2) Update CPGDN-based appearance model using new positive samples $s_{t}^{+}$ and new negative samples $s_{t}^{-}$ if the score of the optimal target state below a threshold *φ*.
**end for**		

Specifically, the main components of the proposed CPGDN-based tracking method are: (i) In an initial frame, we firstly collect some positive samples and negative samples, where positive and negative examples have more than 0.7 and less than 0.5 the Intersection over Union (IoU) overlap ratios with ground-truth bounding boxes. Then, the CPGDN model pre-trained on a large-scale image data set (i.e., Tiny image dataset [56]) is fine-tuned according the positive and negative samples. (ii) In subsequent frames, a set of candidate samples are firstly generated by a prediction function within a particle filtering framework. Then, the final scores for each candidate sample is determined by fusing both scores from the CPGDN model and the edge box-based proposal method. (iii) The optimal target location is determined by the candidate sample with the maximum score. (iv) The CPGDN model is updated if the maximum score of candidate samples below a threshold *φ*. The tracking procedure continues in this iterative fashion until the end of video. Each detained component of the proposed CPGDN-based tracking method is described in the following subsections.

### 2.1 Visual tracking under a particle filtering framework

The proposed CPGDN-based tracking method is carried out using a particle filtering framework [57] which is a technique for implementing recursive Bayesian filter by Monte Carlo sampling. The key idea is to represent the posterior density by a set of random particles/samples with associated weights. The posterior probability can be estimated based on these samples and weights.

Suppose we have an observation of a target object *Y*_*t*_ = {*y*_1_,…,*y*_*t*_} up to the *t*^*th*^ frame, the posterior probability *p*(*x*_*t*_ | *Y*_*t*_) can be calculated by the Bayesian theorem as the following:

$$p(x_{t}|Y_{t})\propto p(y_{t}|x_{t})\int p(x_{t}|x_{t-1})p(x_{t-1}|Y_{t-1})dx_{t-1}$$ (1)

where *p*(*x*_*t*_ | *x*_*t*−1_) is a prediction function, and *p*(*y*_*t*_ | *x*_*t*_) is a likelihood evaluation function which determines the likelihood of observing *y*_*t*_ at state *x*_*t*_. The optimal object state $x_{t}^{*}$ at time *t* can be inferred as follows

$$x_{t}^{*}=\arg\;\max_{x_{t}^{i}}^{i=1,\ldots ,N}\{p(y_{t}^{i}|x_{t}^{i})p(x_{t}^{i}|x_{t-1})\}$$ (2)

where $x_{t}^{i}$ is the *i*^*th*^ sample of the state *x*_*t*_, and $y_{t}^{i}$ is the image observation predicted by $x_{t}^{i}$. In this paper, a target state is denoted by $x_{t}=(l_{t}^{x},l_{t}^{y},w_{t},h_{t})$ where the four parameters are the horizontal coordinate, vertical coordinate, width and height respectively. The prediction function *p*(*x*_*t*_ | *x*_*t*−1_) is modeled by a Normal distribution function, i.e., *p*(*x*_*t*_ | *x*_*t*−1_) = *N*(*x*_*t*_; *x*_*t*−1_, ∑), where Σ is a diagonal covariance matrix whose diagonal elements are the corresponding variances of respective parameters. In order to estimate likelihood of each state *x*_*t*_, we firstly normalize each image patch (i.e., each particle sample) to 32*32 pixels. Then, the likelihood of each particle is calculated based on the CPGDN model, i.e., *p*(*y*_*t*_ | *x*_*t*_) = *d*_*t*_, where *d*_*t*_ is an output score estimated from the CPGDN model.

### 2.2 The CPGDN based appearance model

In this section, we address the problem of how to learn a CPGDN based appearance model via jointly feature learning and selection in a unified framework. We construct a two-layer CPGDN model, in which the first layer is composed by convolutional restricted Boltzmann machines (CRBM) and the second layer is composed by convolutional point-wise gated Boltzmanne machine (CPGBM) followed by a full connection layer.

More specifically, we use the proposed CPGDN [55] to extract features of a target object. The key advantages of CPGDN is convolutional architecture and jointly performing the feature learning and selection in a unified framework. Convolutional architecture is good at dealing with spatially correlated data while feature selection can obtain more robust features from complex real-word data. Inspired by these advantages from the CPGDN model, in this paper, we propose a CPGDN-based method to effectively learn the abstract feature to distinguish a target object from the non-target objects.

**Fig 1:**
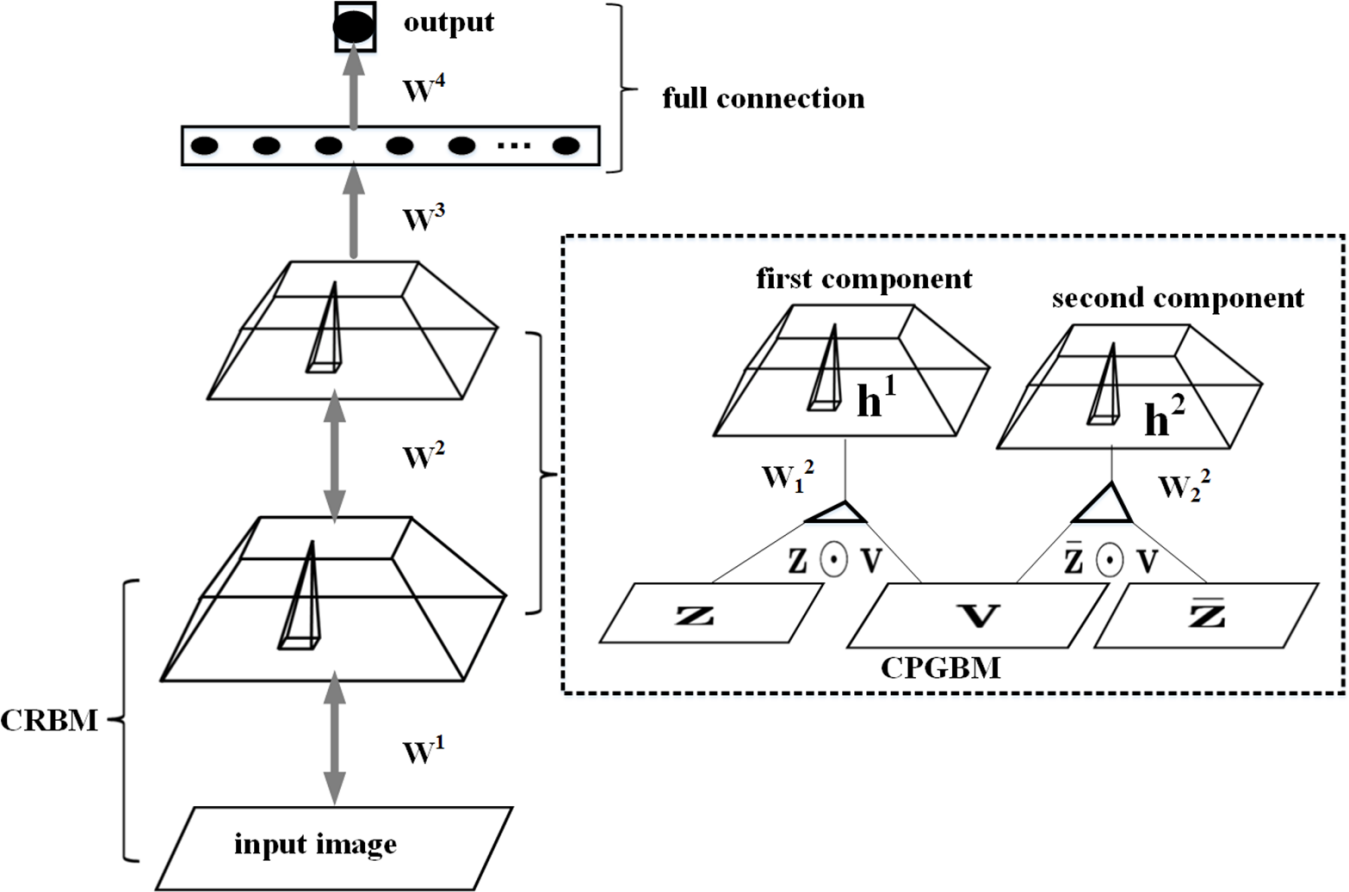
Architecture of the two-layer CPGDN model [55] with a full connection layer. The first layer is CRBM, and the second layer is CPGBM with two mixture components. *z* is a gating mechanism and its value is binary variable. *z* and $\bar{z}$ are complementary, i.e., $\bar{z}=1-z$. We use the first component of CPGBM as the input of a full connection layer.

The CPGDN model is illustrated in Fig 1. Following the notations of Sohn et al. [55], we will briefly review the CPGDN model and focus on how to construct the CPGDN model based appearance model for visual tracking.

#### The generic feature extraction based on CRBM

We use the convolutional RBM with probabilistic max pooling (CRBM) to extract the generic features. Please see [58] for more details about CRBM. The CRBM is composed by a “detection” layer, which is similarly to the convolutional layer of CNN, and a “pooling” layer, which shrink the representation of the detection layer. The CRBM with pooling layer are more robust to small variations. Denote I ∈ ℝ^*N*×*N*×*C*^ as the input image, where *C* denotes the number of input channels (e.g., *C* = 1 for gray images) and *K* denotes the number of filters. *ws*×*ws* as the 2D convolutional filter size. W denotes the square filters of size *s*, i.e., W^*k*,*c*^ ∈ ℝ^*ws*×*ws*^. The operator $\tilde{\text{W}}$ applied in the matrix W denotes the vertical and horizontal flip of the matrix. Please see the experimental section for more details on parameter setting.

#### The semantic feature learning and selection based on CPGBM

Once trained the CRBM, we use the output of pooling layer in CRBM as the input of the CPGBM. Denote z_*m*,*n*_ as the switch units. Denote R as the mixture components. z have the same size with v. Note that all the channels of input v shared the same switch unites z. Intuitively speaking, every channel of input sample shared the same switch unites. Given the other two types of variables, we can compute the conditional probabilities of hidden, switch, and visible units below:

$$P(h_{i,j}^{r,k}=1|\text{v},\text{z})=\sigma \left(\sum_{c}^{}({\tilde{\text{W}}}^{r,k,c}*({\text{z}}^{r}⊙{\text{v}}^{c}){)}_{i,j}+b_{k}^{r}\right)$$ (3)

$$P(z_{m,n}^{r}=1|v,h)=\frac{\exp\;(v_{m,n}^{c}(\sum_{k}{\left(W^{r,k,c}*h^{r,k}\right)}_{m,n}+c_{c}^{r}))}{\sum_{s}\exp\;(v_{m,n}^{c}(\sum_{k}{\left(W^{s,k,c}*h^{s,k}\right)}_{m,n}+c_{c}^{s}))}$$ (4)

$$P(v_{m,n}^{c}=1|\text{h},\text{z})=\sigma \left(\sum_{r}^{}z_{m,n\;}^{r}\left[\sum_{k}^{}{\left({\text{W}}^{r,k,c}*{\text{h}}^{r,k}\right)}_{m,n}+c_{c}^{r}\right]\right)$$ (5)

The operator e denotes an element-wise multiplication between two matrices. The above equations subject to $\sum_{r=1}^{R}z_{m,n}^{r}=1$. Please note that the CPGBM only has convolutional layer and we use the first components of CPGBM as the learnt features.

#### Learning object appearance models from CPGDN

The CPGDN is composed by stacking the CPGBM on the first layer of CRBM. This construction makes sense because the first layer mostly learn the generic features and the higher layer learn semantic features. But, not all semantic features are good for our task and we need typical semantic features for our specific target object. Firstly, we use a large number of images from the Tiny image dataset [56] to offline train the CPGDN model with one fully connection layer. Then we transfer the learned parameters to initial the model used for online tracking. Typically, we can get a ground-truth bounding box of a target object in an initial frame. A warping technology is utilized to generate the positive and negative samples. The positive and negative examples have more than 0.7 and less than 0.5 IoU overlap ratios with the ground-truth bounding boxes. The generated positive and negative examples are used to fine-tune the pre-trained CPGDN model. During the tracking process, we update the CPGDN model using the newly observed target samples when the maximum confidence of all particles/samples is below a pre-defined threshold *φ*.

### 2.3 An edge box-based object proposal method

In this paper, we employ an efficient edge box-based object proposal method [46] for further improving tracking results. The goal is to make our tracker focus on a visual target object.

Specifically, based on a key idea that a bounding box likely contains a visual object if the number of contours wholly enclosed by the bounding box is enough, the edge box-based object proposal method generates a set of object candidates. Firstly, a structured forest based edge detector [59] is used to estimate an edge map for each pixel in an input image. Then, given the extracted edge map, a pool of sampled bounding boxes is generated via a sliding window way. Finally, according to the number of contours wholly enclosed by a bounding box, the score for a bounding box is calculated. For more details, please refer to [46].

### 2.4 The CPGDN-based tracker driven by edge box-based object proposals

In this section, we utilize the edge box-based object proposal method to improve the performance of the proposed CPGDN-based tracker while maintaining its required computational efficiency.

Without loss of generality, suppose we have a set of candidate particles/samples ${\left\{c_{i}\right\}}_{i=1}^{N}$ at the *t*^*th*^ frame. Based on the CPGDN model and the edge box-based object proposal method, we evaluate the likelihood of a candidate sample *c*_*i*_ belonging to the target object. Firstly, we calculate the CPGDN model based score *f*_*T*_(*c*_*i*_) of the candidate sample *c*_*i*_. Then, we calculate the object proposal score *f*_*E*_(*c*_*i*_) of the candidate sample *c*_*i*_ via the edge box-based object proposal method. Consequently, the final score for the candidate sample *c*_*i*_ is calculated as follows.

$$f\left(c_{i}\right)=f_{T}(c_{i})+(f_{E}(c_{i})+\lambda )$$ (6)

The value of parameter *λ* depend on the value of *f*_*E*_(*c*_*i*_) and is calculated as follows.

$$\lambda =\{\begin{array}{l} 0,\;if\;f_{E}\left(c_{i}\right)>0\\ -0.1,\;else, \end{array}$$ (7)

We use the simple yet effective fusing schema to combine the CPGDN-based appearance model with the edge box-based object proposal method. The goal is to make the proposed CPGDN-based tracker focus on a visual target object instead of the non-targets due to the edge box-based object proposals can provide rich information for the proposed CPGDN-based tracker. Consequently, the performance of the proposed CPGDN-based tracker driven by edge box-based object proposals can be greatly improved.

## 3. Experiments

In this section, we introduce extensive experimental results from the proposed CPGDN-based tracker (named CPGDN). Firstly, we describe the setting of our experiments including the implementation details and the evaluation protocol of the CVPR 2013 tracking benchmark [30]. Then, we compare the proposed CPGDN-based tracker with the state-of-the-art trackers on the CVPR 2013 tracking benchmark. Moreover, to verify the effectiveness of the edge box based object proposals method, we compare the standard CPGDN-based tracker with its variant without using the edge box based object proposals method. Finally, we discuss some issues and future work.

### 3.1 Experiment setting

We implement the proposed CPGDN-based tracker in MATLAB. The running speed is about one frame per second on a HP Z800 workstation with an Intel i5-3470 3.20GHz CPU and 22GB RAM. The number of particles are *N* = 600. For feature extraction, each image patch of a target object is warped to 32*32 pixels. In the first layer of CPGDN, we set *ws* = 5 and *K* = 12. To get positive and negative examples, we firstly use a warping technology to the target sample and obtain *N*_1_ = 10 positive samples. Then, we extracted *N*_2_ = 100 negative samples surrounding the target region. The positive and negative examples have more than 0.7 and less than 0.5 IoU overlap ratios with the ground-truth bounding boxes. In the tracking process, once the maximum confidence of all particles/samples in a frame is below a predefined threshold *φ* = *0*.*8*, we update the CPGDN model using the new observed target samples. The same parameters are fixed for all of the experiments.

To extensive evaluate the proposed CPGDN-based tracker, we adopt the widely used one-pass evaluation (OPE) metric from the CVPR 2013 tracking benchmark [30] which contains 50 fully annotated image sequences. The 50 image sequences is tagged by 11 tracking challenging factors, such as illumination variation, scale variation, occlusion, deformation, motion blur, fast motion, in-plane rotation, out-of-plane rotation, out-of-view, background clutter and low resolution. Experimental results are reported using the precision plots and success plots, which rank trackers in terms of center location error at threshold 20 pixels and area under the curve, respectively. Initially, three are 29 trackers are adopted in the benchmark. For more details, please refer to the paper [30]

### 3.2 Comparison with other trackers

#### Quantitative Evaluation

**Fig 2:**
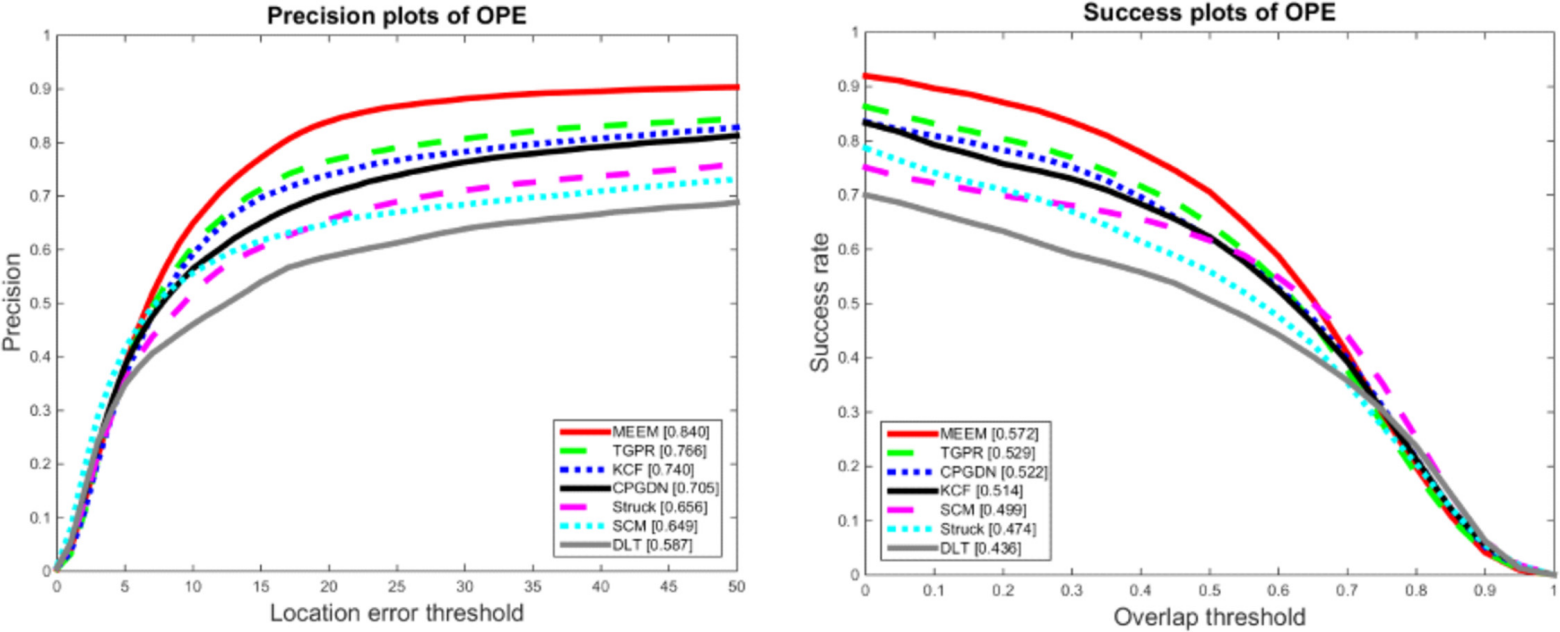
The precision and success plots of quantitative comparison for the 50 sequences in the CVPR2013 tracking benchmark [30].The performance score of each tracker is shown in the legend. The proposed CPGDN-based tracker (named CPGDN) ranks 4th in precision plots and 3th in success plots respectively.

In Fig 2, we show the OPE evaluation results with 29 state-of-art trackers on 50 image sequences [30], where only the top 10 trackers are shown for clarity. In addition, for fair comparisons, we also compare the four recent representative trackers including MEEM [4], KCF [6], DLT [21], and TGPR [60]. Fig 2 shows that the proposed CPGDN-based tracker performs favorably against the state-of-the-art methods on the OPE evaluation metric. More specifically, the proposed CPGDN-based tracker ranks 4th in terms of the precision rate while 3rd based on the success rate. It outperforms DLT by 11.8% in the precision plot and 8.6% in the success plot respectively. In terms of the success plot, the proposed CPGDN-based tracker outperforms KCF. Please note that the key advantage of the proposed CPGDN-based tracker is that it can jointly perform feature selection on raw features through a gating mechanism. Therefore, the proposed CPGDN-based tracker can adaptively focus on the task-relevant patterns (i.e., a target object), while ignoring the task-irrelevant patterns (i.e., the surrounding background of a target object).

#### Attribute-based Evaluation

**Fig 3:**
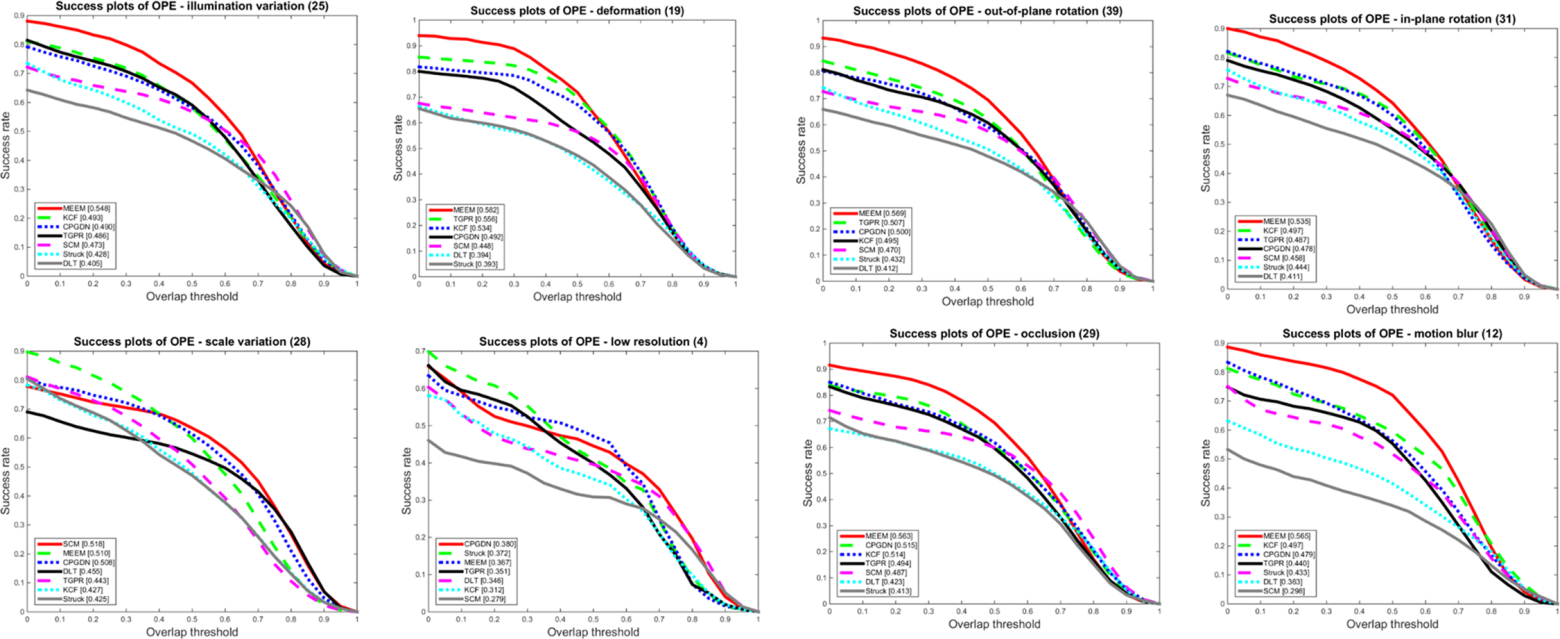
The success plots for eight challenge attributes: illumination variation, deformation, out-of-plane rotation, in-plane rotation, scale variation, low resolution, occlusion, motion blur.

To thoroughly evaluate the performance of the proposed CPGDN-based tracker in various challenging scenes, we summarize the performance based on 11 different factors on 50 image sequences [30]. Due to space limitation, we only show the success plots for eight challenge attributes in Fig 3. As shown in Fig 3, the proposed CPGDN-based tracker performs well against the other methods in almost all tracking attributes.

#### Center Distance Error Evaluation

**Fig 4:**
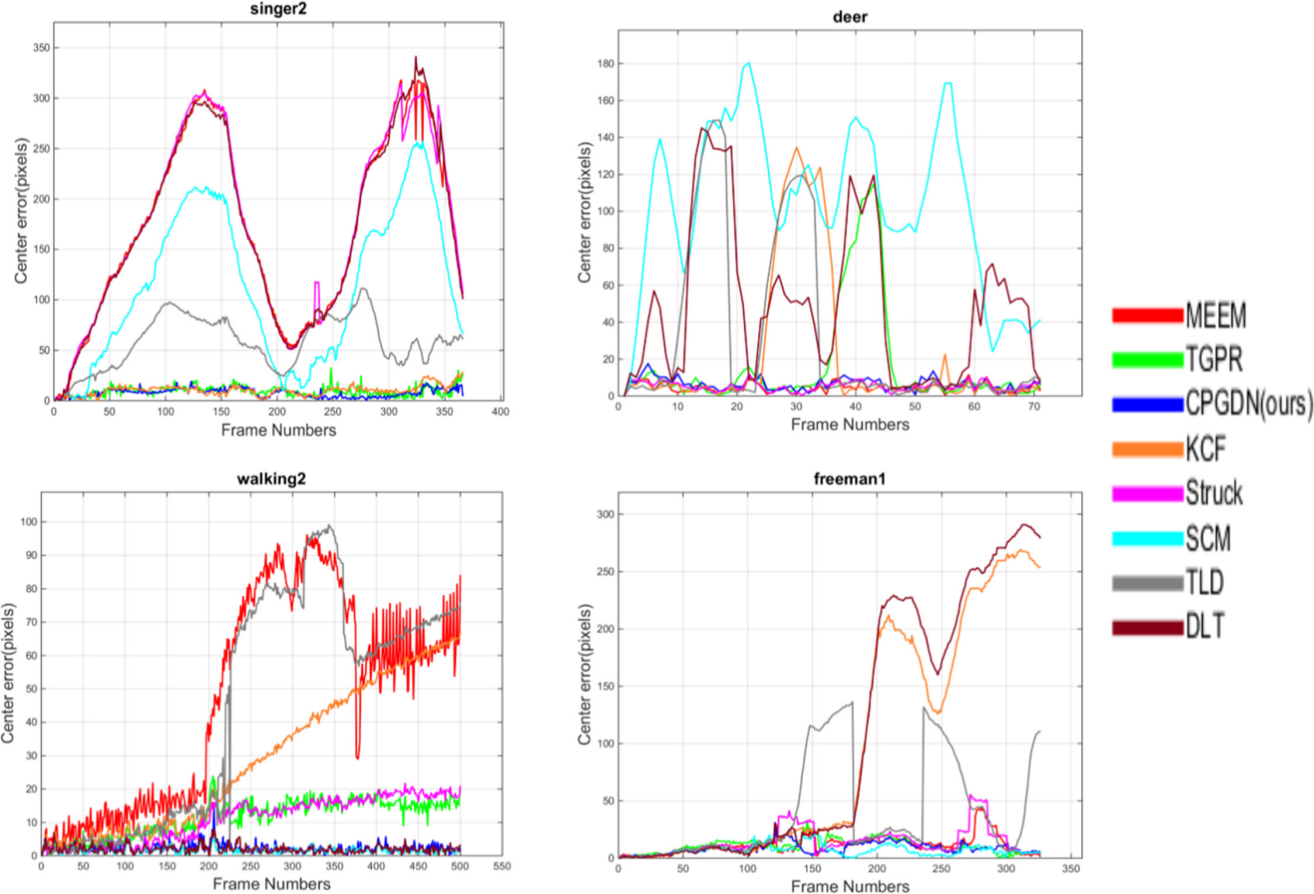
Quantitative comparison on the center distance error per frame for the four image sequences from [30].

In Fig 4, we show the center distance error per frame for the four typical image sequences, i.e., the singer2, deer, walking2, and freeman1 sequence respectively. For presentation clarity, the results by the top 8 trackers are shown. The proposed CPGDN-based tracker can achieve promising results due to jointly learning and selecting robust features via the CPGDN model driven by the edge box based object proposals method.

### 3.3 Efficacy of the edge box based object proposals method

**Fig 5:**
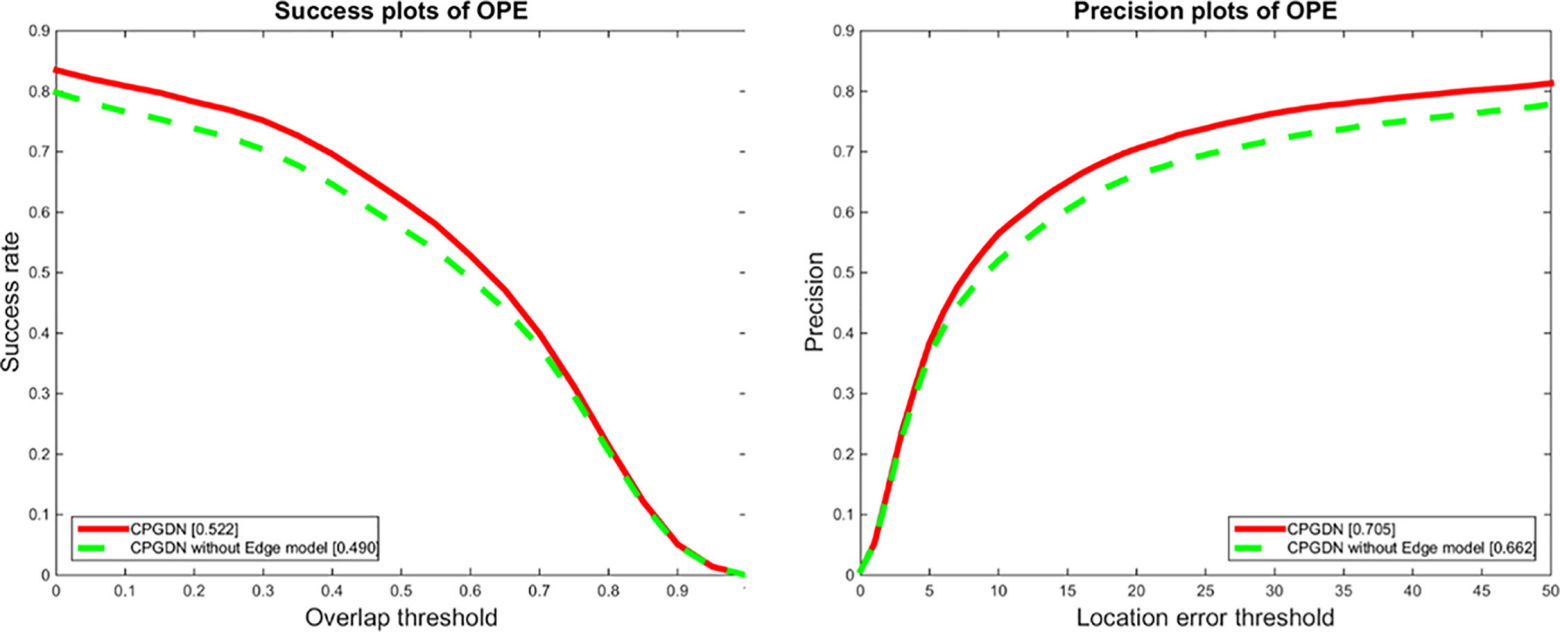
The success plots and precision plots of OPE for the standard CPGDN-based tracker and its variant without the edge box based object proposals method. It is obvious that the proposed CPGDN-based tracker driven by the edge box based object proposal method can achieve promising tracking results.

To verify the effectiveness of proposed edge box based object proposals method for the proposed CPGDN-based tracker, we evaluate the performance of the proposed CPGDN-based tracker without using the edge box based object proposals method. Fig 5 shows the quantitative results on the CVPR 2013 tracking benchmark [30]. As shown in Fig 5, without the edge box based object proposals method, both the precision and success rate reduce to some extent. The precision and success rate reduce about 4.3% and 2.6% respectively. This is consistent to our intuition that a tracker should focus on an object target instead of a non-object target. By combing \the edge box based object proposals method with the CPGDN model, we can effectively alleviate tracker drifting problem to some extent.

### 3.4 Discussion

Although the proposed CPGDN-based tracker has achieved promising results compared with the state-of-the-art trackers, its performance is a bit worse than those of MEEM, TGPR and KCF. In other words, it is still far from perfect. Here we analyze some causes leading to the failure and discuss some possible solutions:

1. The proposed CPGDN-based tracker transfers generic image features that are more robust against variations from pre-training to online tracking. However, due to the powerful invariant feature representation of the CPGDN model, the proposed CPGDN-based tracker may possibly drift when tracking a specific target object which has similar appearance with a distractor.
2. The proposed CPGDN-based tracker is likely to drift when the appearance variations of a target object is huge.

There may be two possible methods to solve the above mentioned issues:

1. In addition to solely relying on the pre-trained CPGDN model, we could build another online appearance model. The online learning-based appearance model can capture the latest appearance variations. These two models can be co-trained to decide the best target state.
2. More effective update strategies could be adopted to improve the tracking results due to an good update strategy can avoid bad samples corrupting the appearance model.

## 4. Conclusion

In this paper, instead of directly using learned features which probably have some noise for tracking, we have proposed a novel visual tracking method via a point-wise gated convolutional deep network (CPGDN) that jointly performs the feature learning and feature selection in a unified framework. Moreover, our model can adaptively select learned features aiming at different target objects. Based on the observation that a visual target should be an object rather than not, we combine an edge box-based object proposal method with the CPGDN based model to effectively alleviate the tracker drifting problem. Extensive experiments on the CVPR2013 tracking benchmark have validated the robustness and effectiveness of the proposed CPGDN-based tracker.
